# An improved saliency model of visual attention dependent on image content

**DOI:** 10.3389/fnhum.2022.862588

**Published:** 2023-02-28

**Authors:** Shabnam Novin, Ali Fallah, Saeid Rashidi, Mohammad Reza Daliri

**Affiliations:** ^1^Faculty of Biomedical Engineering, Amirkabir University of Technology (AUT), Tehran, Iran; ^2^Faculty of Medical Sciences and Technologies, Science and Research Branch, Islamic Azad University, Tehran, Iran; ^3^Neuroscience and Neuroengineering Research Laboratory, Biomedical Engineering Department, School of Electrical Engineering, Iran University of Science and Technology, Tehran, Iran; ^4^School of Cognitive Sciences (SCS), Institute for Research in Fundamental Sciences (IPM), Tehran, Iran

**Keywords:** visual attention, saliency model, medium-level features, center-surround difference map, eye fixations

## Abstract

Many visual attention models have been presented to obtain the saliency of a scene, i.e., the visually significant parts of a scene. However, some mechanisms are still not taken into account in these models, and the models do not fit the human data accurately. These mechanisms include which visual features are informative enough to be incorporated into the model, how the conspicuity of different features and scales of an image may integrate to obtain the saliency map of the image, and how the structure of an image affects the strategy of our attention system. We integrate such mechanisms in the presented model more efficiently compared to previous models. First, besides low-level features commonly employed in state-of-the-art models, we also apply medium-level features as the combination of orientations and colors based on the visual system behavior. Second, we use a variable number of center-surround difference maps instead of the fixed number used in the other models, suggesting that human visual attention operates differently for diverse images with different structures. Third, we integrate the information of different scales and different features based on their weighted sum, defining the weights according to each component's contribution, and presenting both the local and global saliency of the image. To test the model's performance in fitting human data, we compared it to other models using the CAT2000 dataset and the Area Under Curve (AUC) metric. Our results show that the model has high performance compared to the other models (AUC = 0.79 and sAUC = 0.58) and suggest that the proposed mechanisms can be applied to the existing models to improve them.

## Introduction

The brain uses attention mechanisms to process a massive amount of visual information, selecting the significant items while ignoring the unimportant ones (Carrasco, 2011). Human visual attention acts based on two mechanisms of bottom-up and top-down interacting through various brain areas (Doricchi et al., 2010; Dehghani et al., 2020). Bottom-up attention functions in an involuntary and fast way based on the psychophysical properties of the scene, while top-down attention functions in a voluntary and slow way based on pre-defined goals (Connor et al., 2004).

Many computational models have been presented for visual attention. Few have modeled top-down visual attention (Deco and Zihl, 2001; Denison et al., 2021; Novin et al., 2021), while most of them have modeled bottom-up visual attention, as its mechanisms are better understood (Zhang et al., 2008). Visual attention models that aim to obtain the saliency of a scene are called saliency models. Saliency refers to how some parts of a scene stand out compared to their surroundings in our visual perception (Borji and Itti, 2013). Saliency models are typically evaluated based on how well they can predict human attentional gaze while viewing different images (Krasovskaya and MacInnes, 2019; Ullah et al., 2020).

Many different approaches have been proposed for saliency detection. Some of them operate based on objects' information in the image (Liu et al., 2010). The Bayesian-based models combine the prior constraints with scene information in a probabilistic way to find the rare features (Zhang et al., 2008). Some models determine the most informative parts of the scene as salient (Bruce and Tsotsos, 2005). The frequency-based models determine the image regions with unique frequencies as salient (Hou and Zhang, 2007). The other category is learning-based models that train the model with human data (Judd et al., 2009).

Recently, many saliency models have focused on using learning algorithms. Although learning algorithms have significantly improved the performance of saliency models (Kummerer et al., 2017), they operate as a black box rather than including particular mechanisms to address the behavior of visual attention. In addition, they usually require many data to train the model. Here, we present a saliency model to investigate some of the mechanisms of bottom-up attention behavior. The missing understanding of the mechanisms of bottom-up attention and decreasing focus on investigating these mechanisms motivated us to do this study.

There are still open problems in the bottom-up aspect of the saliency models, addressed in recent studies (Zhang and Sclaroff, 2016; Ayoub et al., 2018; Wang et al., 2018; Molin et al., 2021). These problems include determining the appropriate features to apply (Kummerer et al., 2017; Narayanaswamy et al., 2020), establishing center-surround differences in the image (Zhang and Sclaroff, 2016; Ayoub et al., 2018), and integrating multiple features and scales (Jian et al., 2019; Narayanaswamy et al., 2020) to obtain salient areas in the image.

Here, we make some improvements based on the existing knowledge about the visual system. (1) We investigate some missing features to be considered. (2) How can we integrate the image information better for creating the saliency map. (3) How can we improve the mechanism of calculating center-surround differences in an image by adapting it to the image structure. In the following, we review previous studies in line with our focus on these improvements.

The rest of our paper is structured as follows. In Section Related study, we review the related studies and end with the contributions of our model. Afterward, in Section Methods, we explain our model. In Section Results, we show the results and compare the model's performance with other models. In Section Discussion, we discuss the results. Finally, in Section Conclusion, we make conclusions.

## Related study

### Itti's base model of visual attention

The base model of saliency was presented by Itti et al. (1998), commonly known as the Itti model. It was known as a pioneer and benchmark model because it is based mainly on the behavior of the human early visual system. The Itti model predicts the saliency map by applying the center-surround (C–S) mechanism to three features of intensity, color, and orientation that are known to be critical low-level features in bottom-up attention (Wolfe and Horowitz, 2004; Frintrop et al., 2015). The C–S difference maps between different scales are calculated for each feature map to simulate visual system behavior, which is attracted by areas that are more distinct from their surroundings (Casagrande and Norton, 1991). Then, the C–S difference maps are combined across various scales and features to obtain the saliency map, which is a topographic representation of saliency for each pixel in an image.

Later, many models made improvements to different steps of the base model of Itti (Borji and Itti, 2013). In the following, we review some of these studies.

### Decomposing images into multi scales using wavelets

In most models, the image is decomposed to multiple scales using the classic Gaussian filter, while here, we apply wavelet-based decomposition. Recently, using the wavelet transform (WT) in saliency models has been shown to be beneficial (Murray et al., 2011; Imamoglu et al., 2013; Ma et al., 2015). WT has the advantage of simultaneously providing spatial and frequency information at each image scale (Murray et al., 2011). Also, it extracts oriented details of the image in horizontal, vertical, and diagonal dimensions at each scale (Imamoglu et al., 2013). WT is a powerful tool for spatial-frequency (Antonini et al., 1992) and time-frequency analysis (Sadjadi et al., 2021). In a spatial-frequency analysis, WT decomposes the image into multiple levels by iteratively performing horizontal and, subsequently, vertical sub-sampling on it through a set of filters (Antonini et al., 1992).

Murray et al. (2011) model the center-surround effect based on contrast energy ratios at the central and surrounding regions using WT. Then they weigh the scales of the wavelet pyramid by a contrast sensitivity function (CSF). Finally, they obtain the saliency map by combining the inverse WT of the weighted maps. In Murray et al. (2011), the computation of the saliency map is mainly based on local contrasts in the image. Imamoglu et al. (2013) obtained the feature maps by applying WT until the coarsest possible level. Their model obtains the general saliency map by modulating the locations' local saliency with their global saliency. Abkenar and Ahmad (2016) proposed a saliency model according to the wavelet coefficients calculated for superpixels to make the model applicable to more complex images.

### Using multi-scale features to create C–S difference maps

The experiment of Bonnar et al. (2002) indicates that the perceived information of an image can be present at different scales. Hence, integration of the information of different scales is needed to obtain the final saliency. The question is how many different scales of C–S difference maps we need to integrate. Previous models have used different numbers, and no evaluation has been done on what can be a proper number to choose. The base model of Itti et al. (1998) employs 6 C–S difference maps created for low-level features. Zhao and Koch (2011) use a similar model to Itti's, adding the face feature. In Zhang et al. (2008), Goferman et al. (2012), and Ma et al. (2013), four scales of feature maps are used. The authors Kruthiventi et al. (2017) and Qi et al. (2019) proposed a multi-scale convolutional neural network (CNN), where each CNN is trained to obtain the salient locations at a particular scale. Vig et al. (2014) combine various models to take advantage of each one. The authors discuss that one of the factors that makes the models different is the scale on which they perform.

In contrast to the common strategy of using a fixed number of scales, we discuss that images with different structures may require a different number of scales to present the saliency of objects in the image.

### Integration of information to create the saliency map

Conventionally, in many models, the saliency map is obtained by linearly combining different scales and features (like Itti et al., 1998; Borji, 2012; Goferman et al., 2012; Imamoglu et al., 2013; Wei and Luo, 2015; Zeng et al., 2015). The objects produce different amounts of saliency at different scales, depending on their size, details, etc. Similarly, different features are not equally relevant to describe an object's saliency. Therefore, a linear combination of different scales and features may not produce results fitting to human data.

Some models made improvements by applying different methods than linear combinations. Itti and Koch (2001) compared four different methods for normalizing the feature maps based on their distributions. Murray et al. (2011) weigh the scales using the contrast sensitivity function proposed by Otazu et al. (2010) and fitting it to psychophysical data. They obtain the final saliency map by combining the Euclidean norm of the maps of different channels. Narayanaswamy et al. (2020) prioritize the feature maps at multiple levels based on the 2D entropy of the maps. Then they calculate the model score for several channel combinations to find the informative channels. Zhao and Koch (2011) set the weights of feature maps based on learning different datasets using the least square method. Borji et al. (2011) use an evolutionary optimization method to set the weights of the scales and feature maps so that they lead to maximum scores and minimum processing costs. Singh et al. (2020), in their optimization method, define an objective to increase the activity in the saliency map at the location of a salient object and decrease the activity in the background.

Here, we make some improvements to previous models to consider some missing mechanisms. Our contributions are listed below:

- We propose that in addition to the commonly used low-level and high-level features, the medium-level features based on the combination of orientations and colors play a role in bottom-up attention.
- We apply a weighting method for across-scale and across-feature integration, presenting the image's local and global saliency. Furthermore, we compare the weighting method for integrating features' conspicuity maps with the method of calculating their maximum.
- We propose using a variable number of center-surround difference maps depending on the structure of the images. This is an important part of our contributions.

## Methods

**Figure 1:**
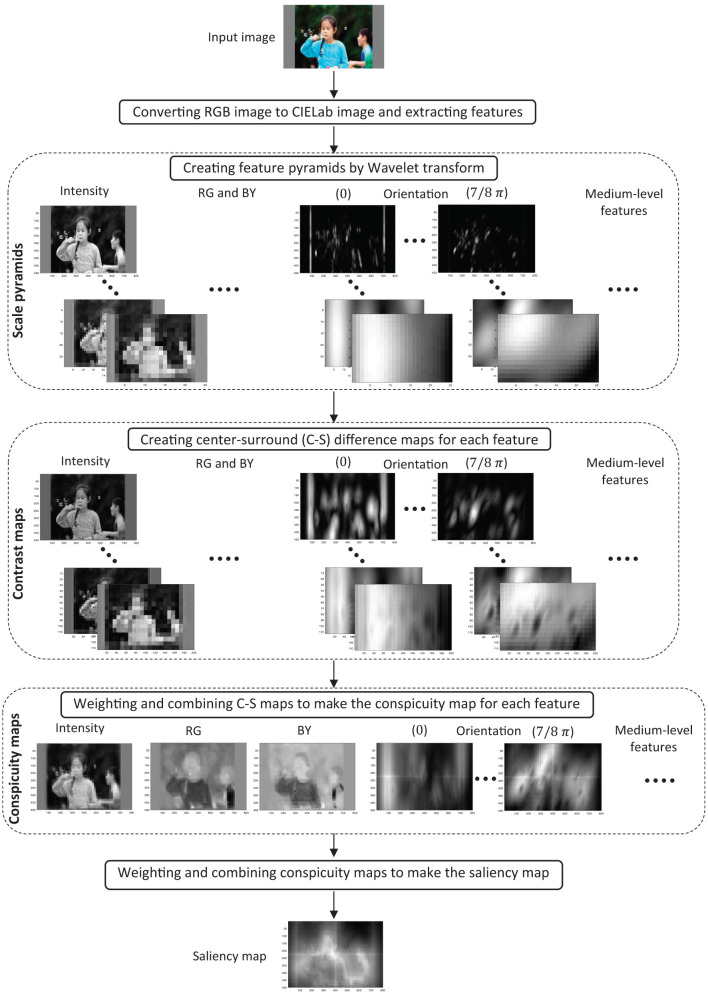
The proposed model structure. The outputs of each layer are shown for a sample image. RG and BY denote red-green and blue-yellow channels, respectively. For more details, refer to the main text.

The block diagram of the proposed model is shown in Figure 1. The input of the model is an RGB image. The model consists of five layers, where each layer's output is shown for a sample image in the figure. In the first layer, the visual features of the image are extracted. In the second layer, the scale pyramids are acquired for each feature using wavelet transform. In the third layer, the difference between the high and low-resolution pyramid levels is calculated for each feature in the specified levels to make the center-surround difference maps. In the fourth layer, C–S difference maps are integrated for each feature to create the feature's conspicuity map. Finally, in the fifth layer, the conspicuity maps of different features are combined to make the final saliency map. In the following, the computations within the layers are described in detail.

### Layer I–Extracting the features

First, the input RGB images are converted to CIELab color space. Compared to RGB used in Itti's base model and many other models, CIELab is a perceptually uniform color space (Frintrop, 2006; Borji, 2012). The CIELab color space consists of L, a, and b channels, representing luminance, the green-red opponent colors, and the blue-yellow opponent colors, similar to human color perception (Frintrop, 2006; Borji, 2012; Ma et al., 2013). The intensity, color, and orientation features are extracted from the CIELab image.

The intensity feature is computed by Equation (1), where R, G, and B, respectively, stand for the red, green, and blue channels of the RGB image.


(1) $$\begin{array}{l} Intensity=0.2989\times R+0.5870\times G+0.1140\times B \end{array}$$

Since a and b channels in CIElab space are based on the opponent color model of human visual cells (Frintrop, 2006), we use a and b channels, respectively, as green-red and blue-yellow color opponency features to account for this behavior of visual cells.

A set of 8 orientation features is obtained by convolving the intensity map in (1) with a set of eight Gabor filters at the wavelength of 10 and orientations of $\left\{0,\frac{1\pi }{8},\;\frac{2\pi }{8},\;\frac{3\pi }{8},\;\frac{4\pi }{8},\;\frac{5\pi }{8},\;\frac{6\pi }{8},\;\frac{7\pi }{8}\;\right\}$.

Previous models have used various low-level and high-level features, and it is still under debate how much and in which image areas each of these two kinds of features contribute to predicting the human gaze data (Kummerer et al., 2017). Here, in addition to the low-level features mentioned above, we consider the role of medium-level features. The neurophysiological findings of Ts'o and Gilbert (1988) and the study of Koene and Zhaoping (2007) provide evidence for the existence of primary visual cells driven by saliency according to the conjunction of color and orientation. Based on these studies, we suggest that medium-level features based on the combination of orientations and colors also play a role in bottom-up visual attention. A set of eight medium-level features is obtained by convolving the red-green channel with a set of eight Gabor filters at the wavelength of 10 and orientations of $\left\{0,\frac{1\pi }{8},\;\frac{2\pi }{8},\;\frac{3\pi }{8},\;\frac{4\pi }{8},\;\frac{5\pi }{8},\;\frac{6\pi }{8},\;\frac{7\pi }{8}\right\}$. Similarly, a set of eight medium-level features is obtained by convolving the blue-yellow channel with the same Gabor filters.

### Layer II–Creating feature pyramids

In most models, the pyramids are built using Gaussian decomposition. Here, according to the advantages described in Section Decomposing images into multi scales using wavelets, wavelet decomposition is applied. The wavelet function is selected based on its properties: orthogonality, symmetry, compact support, and regularity. The Daubechies and Symlet wavelets are more frequently used in saliency studies (Jian et al., 2015; Zhu et al., 2019). Here, we choose the Symlet wavelet having more symmetry, which avoids phase distortion. We use Symlet of order 4 (sym4), which has also been used in some other studies (Zhang et al., 2011; Ghasemi et al., 2013). Using a sym4 wavelet, a pyramid of eight scales is obtained for each feature.

### Layer III—Creating center-surround difference maps

In order to obtain areas with high contrast compared to their surroundings, the difference between different scales of the image is calculated. For each feature pyramid, we calculate the difference between the scales of low numbers and the scales of two and three higher numbers. The lower scales with a high resolution and the higher scales with a low resolution can be considered respectively as the center and surround areas to compute the center-surround difference maps for each feature, as described in Equation (2).


(2) $$\begin{array}{l} I_{c,s}=|I_{c}⊝I_{s}|\\ RG_{c,s}=|RG_{c}⊝RG_{s}|\\ BY_{c,s}=|BY_{c}⊝BY_{s}|\\ O_{c,s,\theta }^{I}=|O_{c,\theta }^{I}⊝O_{s,\theta }^{I}|\\ O_{c,s,\theta }^{RG}=|O_{c,\theta }^{RG}⊝O_{s,\theta }^{RG}|\\ O_{c,s,\theta }^{BY}=|O_{c,\theta }^{BY}⊝O_{s,\theta }^{BY}| \end{array}$$

Where *c* refers to the center part represented by lower scales and *s* refers to the surrounding region represented by higher scales. θ denotes the orientation of the Gabor function. The symbol ⊝ denotes the C–S difference operator, where the high-scale image is interpolated to the size of the low-scale image, and then the two images are subtracted.

Most saliency models use a fixed number of C–S difference maps. Here, we discuss that the human visual system does not attend to the contrasts in the same way for all images, containing different amounts of detailed and coarse content. Hence, different numbers of contrast maps are required for different images to model visual attention. Several factors, such as the image's crowdedness, the size of the objects, and the variety of each feature, may determine the amount of detailed and coarse content in an image that can affect the required number of contrast maps. We investigated our hypothesis by applying 4, 6, and 10 contrast maps for each feature. The numbers 4, 6, and 10 are chosen exemplary to refer to, respectively, a low, medium, and a high number of contrast maps. We will compare all three groups of results with the human data in the results section. For the final results, we will compute the model's performance based on the maximum value between the results for using 4, 6, and 10 contrast maps. The values of *c* and *s* in Equation (2), denoting the scale number, are defined as described in equations (3–6).


(3) $$\begin{array}{l} \text{Using 4 contrast maps}:c\epsilon \left\{1,2\right\} \end{array}$$

(4) $$\begin{array}{l} \text{Using 6 contrast maps}:c\epsilon \left\{1,2,3\right\} \end{array}$$

(5) $$\begin{array}{l} \text{Using 10 contrast maps}:c\epsilon \left\{1,2,3,4,5\right\} \end{array}$$

(6) $$\begin{array}{l} \text{The s values are defined as }s=c+\delta ,\delta \epsilon \left\{2,3\right\} \end{array}$$

In the case of using *cϵ* {1, 2}, we would obtain 4 contrast maps for each feature. Thus, it would yield 27 × 4 = 108 contrast maps in total. In the same way, in the case of using *cϵ* {1, 2, 3}, we would obtain 27 × 6 = 162 contrast maps in total, and in the case of using *cϵ* {1, 2, 3, 4, 5}, we would obtain 27 × 10 = 270 contrast maps in total. The results for a sample input image are shown in the third layer of the model in Figure 1 by applying 6 contrast maps as an example.

In Table 1, the calculations in equations 2–6 are detailed to show how the difference between scale numbers is calculated in the three approaches of using 4, 6, and 10 contrast maps.

**Table 1:** A detailed description of the calculations in equations 2–6 for obtaining C–S difference maps for each feature.

**Approach**	**Difference between scale numbers c and s, calculated for obtaining C–S maps**
Using 4 contrast maps	1⊝3, 1⊝4, 2⊝4, 2⊝5
Using 6 contrast maps	1⊝3, 1⊝4, 2⊝4, 2⊝5, 3⊝5, 3⊝6
Using 10 contrast maps	1⊝3, 1⊝4, 2⊝4, 2⊝5, 3⊝5, 3⊝6, 4⊝6, 4⊝7, 5⊝7, 5⊝8

The symbol ⊝ denotes the center-surround difference operator. In each row, the numbers written before and after ⊝ refer, respectively, to parameters c and s.

As it is seen in equations (3–6) and Table 1, using each number of C–S maps yields the contrast between specific scales. The lower scales (close to scale 1) contain the fine (detailed) content of the image, and the higher scales (close to scale 8) contain the coarse (rough) content of the image. As a result, we can say that using 4 C–S maps yields the contrasts calculated as very detailed scales (1, 2) minus detailed scales (3) and also minus middle-coarse (4, 5) scales. Using 6 C–S maps yields the same contrasts as 4 C–S maps. In addition, it yields the contrasts computed as detailed scales (3) minus middle-coarse (5, 6) scales. Using 10 C–S maps yields the same contrasts as 6 C–S maps. In addition, it yields the contrasts computed as middle-coarse scales (4, 5) minus coarse scales (7, 8).

### Layer IV–Obtaining conspicuity maps

In this step, the contrast maps of each feature are normalized between [0, 1], and then they are combined using a weighted summation to construct the conspicuity map for the related feature. Following the behavior of the visual system, we use the contrast sensitivity function to calculate the weight of different spatial information. The Contrast sensitivity function shows how much the human visual system is sensitive to contrast changes in a scene in different spatial frequencies. We define the weight of each contrast map dependent on the importance of its spatial frequency information. We use the widely accepted CSF model proposed by Mannos and Sakrison (1974), described by Equation (7), which is also used in some other saliency models (Buzatu, 2012; Wang et al., 2018); however, they do not apply it for weighting contrast maps. The normalized contrast maps are transformed to the frequency domain by Fourier transform (Brigham and Morrow, 1967). Then, the contrast sensitivity function *C*(*f*) is calculated by Equation (7).


(7) $$\begin{array}{l} C\left(f\right)=\left[0.0499+0.2964\times f\right]\times \exp\left[-{\left(0.114\times f\right)}^{1.1}\right] \end{array}$$

Where f is the spatial frequency.

We obtain two conspicuity maps for each feature based on its contrast maps' global and local weighting. For the global weighting, the weight of the normalized contrast map number *i* [termed as ω_*i*_ in Equation (8)] is calculated by averaging *C*_*i*_(*f*) over the map. Then, the weighted and normalized sum of the contrast maps for a specific feature *j* is calculated to get the conspicuity map for feature *j* [termed as *Global conspicuity*_*j*_ in Equation (9)].


(8) $$\begin{array}{l} \omega_{i}=mean\left(C_{i}\left(f\right)\right) \end{array}$$

(9) $$\begin{array}{l} {Global\;conspicuity}_{j}=\frac{\sum_{i=1}^{N}\left(\omega_{i}\times {Contrast\;map}_{j,i}\right)}{\sum_{i=1}^{N}\left(\omega_{i}\right)} \end{array}$$

Where *i* refers to the index of the contrast map for a specific feature, *j* refers to the index of the feature, and *N* = 4, 6, and 10, respectively, for the case of using 4, 6, and 10 contrast maps.


$$\begin{array}{l} j\epsilon \left\{I,RG,BY,O_{\theta }^{I},O_{\theta }^{RG},O_{\theta }^{BY}\right\},\\ \theta \epsilon \left\{0,\frac{1\pi }{8},\;\frac{2\pi }{8},\;\frac{3\pi }{8},\;\frac{4\pi }{8},\;\frac{5\pi }{8},\;\frac{6\pi }{8},\;\frac{7\pi }{8}\;\right\} \end{array}$$

For the local weighting, the *C*(*f*) matrix is multiplied pixel-wise in the associated normalized contrast map. Then the weighted and normalized sum of the contrast maps for each feature is calculated to get its conspicuity map [termed as *Local conspicuity*_*j*_ in Equation (10)].


(10) $$\begin{array}{l} {Local\;conspicuity}_{j}=\frac{\sum_{i=1}^{N}\left(C_{i}\left(f\right).{Contrast\;map}_{j,i}\right)}{\sum_{i=1}^{N}mean\left(C_{i}\left(f\right)\right)} \end{array}$$

The global and local conspicuity maps of each feature are combined according to Equation (11) to yield its final conspicuity map. The weights α = 0.95 and β = 0.05 in Equation (11) are set and optimized by trial and error to better fit the results to the human data in MIT dataset provided in Judd et al. (2009). The conspicuity maps for a sample input image are shown in the fourth layer of the model in Figure 1 for the case of applying six contrast maps as an example.


(11) $$\begin{array}{l} {Conspicuity}_{j}=\alpha \times {Global\;conspicuity}_{j}\\ +\beta \times {Local\;conspicuity}_{j} \end{array}$$

### Layer V–Obtaining saliency map

The saliency map represents the saliency degree at every location of the image as the level of brightness in a grayscale image. The final saliency map is obtained by combining the conspicuity maps of different features. Here, we compare four different methods for integrating various features to find the best method among them. These four integration methods that are described below were developed based on a preliminary study testing 12 different variations of the presented integration methods.

#### Four investigated methods for integrating features

##### First method

In the first method, we obtain the saliency values by calculating the maximum value among the conspicuity maps of different features at each pixel using Equation (12). The reasoning is that we use the values that have more potential to make high conspicuity at each location. This strategy would be a more pixel-wise and local strategy to predict human attentional focus.


(12) $$\begin{array}{l} Sal\;map=Max\left(Conspicuity_{j}\right) \end{array}$$

$$\begin{array}{l} j\epsilon \left\{I,RG,BY,O_{\theta }^{I},O_{\theta }^{RG},O_{\theta }^{BY}\right\},\\ \theta \epsilon \left\{0,\frac{1\pi }{8},\;\frac{2\pi }{8},\;\frac{3\pi }{8},\;\frac{4\pi }{8},\;\frac{5\pi }{8},\;\frac{6\pi }{8},\;\frac{7\pi }{8}\;\right\} \end{array}$$

In Equation (12), the conspicuity maps of orientation features of *O*^*I*^, *O*^*RG*^, and *O*^*BY*^ are obtained by adding the conspicuity maps of the associated feature for 8 orientations and then scale normalizing between [0, 1].

##### Second method

In the second method, we combine the conspicuity maps using a weighted summation. The weight of each conspicuity map is defined based on the difference between the global maximum and average of local maxima in the related map as below. This method is used in a similar way in some other studies (Itti and Koch, 2001; Frintrop et al., 2007).

First, all conspicuity maps are normalized between [0, 1]. Second, we find the local maximum areas in each conspicuity map *j*. Then we calculate the global maximum value (*Max*_*j*_) and the average value (*mean*_*j*_) among the local maxima except *Max*_*j*_. The weights of the conspicuity maps are calculated by Equation (13).


(13) $$\begin{array}{l} w_{j}=|Max_{j}-mean_{j}| \end{array}$$

Where *w*_*j*_ is the weight of the conspicuity map of feature *j*, and


$$\begin{array}{l} j\epsilon \left\{I,RG,BY,O_{\theta }^{I},O_{\theta }^{RG},O_{\theta }^{BY}\right\},\\ \theta \epsilon \left\{0,\frac{1\pi }{8},\;\frac{2\pi }{8},\;\frac{3\pi }{8},\;\frac{4\pi }{8},\;\frac{5\pi }{8},\;\frac{6\pi }{8},\;\frac{7\pi }{8}\;\right\} \end{array}$$

The amount of difference between the global maximum and averaged local maxima in Equation (13) represents the amount of contrast that the related conspicuity map can make to draw attention.

Third, the final saliency map, *Sal map*, is obtained by Equation (14) as the weighted sum of the conspicuity maps of various features.


(14) $$\begin{array}{l} Sal\;map=\frac{\sum_{j}\left(w_{j}\times Conspicuity_{j}\right)}{\sum_{j}\left(w_{j}\right)} \end{array}$$

##### Third method

In the third method, we obtain the weights directly by calculating the difference between the global maximum and the local maxima, in contrast to the averaged local maxima used in the second method. Thus, the weights in the third method would be as matrices and multiplied point-wise in the conspicuity maps. The weights in the second method rely more on the global saliency that each conspicuity map can produce, and the weights in the third method rely more on the local saliency of each conspicuity map.

In the third method, we calculate the values of the conspicuity map *j* at the location of local maxima to produce the matrix *LocalMax*_*j*_. The weights of the conspicuity maps are calculated by Equation (15).


(15) $$\begin{array}{l} W_{j}=|Max_{j}-LocalMax_{j}| \end{array}$$

Where *W*_*j*_ is the weight of the conspicuity map of feature *j*.

The final saliency map, *Sal map*, is obtained by Equation (16) as the weighted sum of the conspicuity maps of different features.


(16) $$\begin{array}{l} Sal\;map=\frac{\sum_{j}\left(W_{j}.Conspicuity_{j}\right)}{\sum_{j}\left(W_{j}\right)} \end{array}$$

##### Fourth method

According to comparing the results of the second and third methods, both methods had almost the same overall performance for the MIT dataset. However, for different images, one of the two methods performed slightly better than the other. This indicates that depending on the structure of the image, the global or local weighting of conspicuity maps may be more efficient. Based on this, in the fourth method, we add linearly the saliency maps obtained by means of the second and third methods to incorporate the properties of both methods. The results showed a little better performance for the fourth method compared to the second and third methods.

We use the fourth method as the final approach for combining different features based on weighted summation. The saliency map for a sample input image is shown in the last layer of the model in Figure 1 based on the fourth method for obtaining the saliency map.

Furthermore, in the results section, we will compare the results of the first method, which is based on maximizing the conspicuity maps, with the fourth method, which is based on the weighted summation of the maps. The comparison results show that the weighting method leads to better results.

### Different versions of the model

We define three versions of our model to investigate the effect of each improvement that we made.

**Proposed model 1** refers to the version in that we used a fixed number (6) of C–S difference maps for each feature, and we did not apply the medium-level features. The model was chosen to contain six C–S maps to be in line with the base model of Itti et al. (1998). In this model version, we investigate the effect of applying our approaches for integrating the information of different scales (described in Section Layer IV–obtaining conspicuity maps) and features (described in Section Layer V–obtaining saliency map). This model version is considered as a baseline to be compared with proposed model 2.

**Proposed model 2** refers to the version in which we extended proposed model 1 by applying the medium-level features (described in Section Layer I–Extracting the features) to investigate the effect of applying them. This model version is considered as a baseline to be compared with proposed model 3.

**Proposed model 3** refers to the full version of the model in that we extended proposed model 2 by applying different numbers of 4, 6, and 10 for the number of C–S difference maps (described in Section Layer III–creating center-surround difference maps), and we calculated the maximum score among the scores of using 4, 6, and 10 maps. In this model version, we investigate the effect of applying variable numbers for C–S difference maps.

### Dataset overview and evaluation metrics

We evaluated our model using the CAT2000 dataset (Borji and Itti, 2015; Bylinskii et al., 2019), which includes 24 subjects' eye tracking data on 2,000 images classified into 20 different categories of 100 images. The images and fixation maps in the dataset have a size of 1,080 × 1,920. We resize the images to 450 × 800. The CAT2000 dataset contains images of different types, including art, cartoons, black white, indoor, outdoor, low resolution, noisy, objects, and outdoor natural. This large variety provides a proper way to validate the model's ability to predict human data related to images with different structures.

The resulting saliency maps were resized to the size of data fixation maps (1,080 × 1,920). The model's performance in predicting human fixations was evaluated based on AUC (Area Under Curve) metric (Bylinskii et al., 2019), which shows the area under ROC (Receiver Operating Characteristic) curve. The ROC curve plots the true positive rate vs. the false positive rate based on comparing the model's fixations to the dataset fixations. A higher AUC denotes a higher performance. According to previous studies that reviewed the models using different evaluation metrics, AUC is the most common metric (Kummerer et al., 2018; Bylinskii et al., 2019). Our goal is to investigate the effect of improvements we made on the model. For this purpose, we found the location-based metric of AUC sufficient to evaluate the results considering that we will compare each improved model version with its lower model version based on this metric. In addition, to compare our model to the other models, we also used the shuffled AUC (sAUC) metric (Bylinskii et al., 2019). The sAUC is similar to AUC with the difference that for AUC, the negative set is selected uniformly at random from the fixation map of the image, while, for sAUC, the negative set is selected from fixation maps of the other images sampled from the dataset. The sAUC is designed to penalize the models that explicitly apply center bias (Zhang et al., 2008). Since some of the to-be-compared models apply center bias to their results, we used sAUC to compare the models better. We used the AUC-Borji and sAUC algorithms presented in Borji et al. (2013).

## Results

### Visualizing the results of the model on sample images

**Figure 2:**
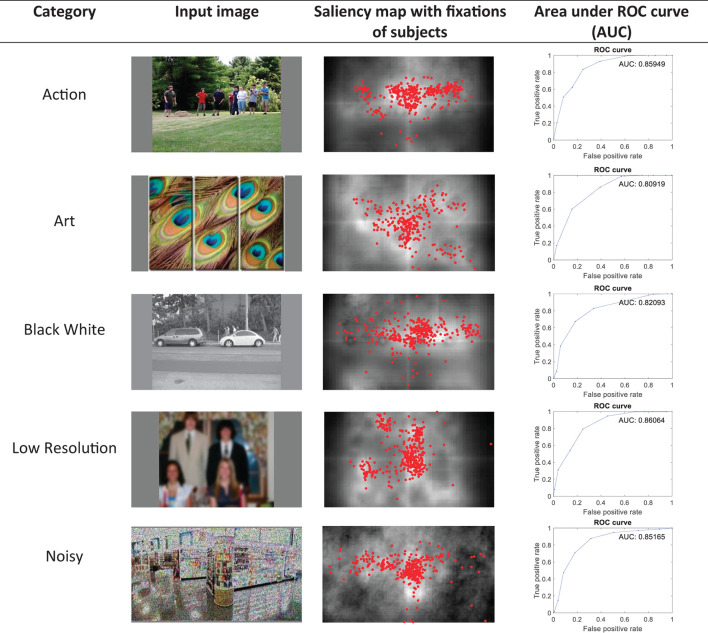
Saliency map results and the AUC scores of the model for sample images of the CAT2000 dataset from various categories of Action, Art, Black White, Low Resolution, and Noisy.

In Figure 2, the saliency map results and the AUC scores of the model for sample images from various categories are shown to show the model's performance for different image structures and conditions. The results are obtained using the fourth method of combining conspicuity maps described in Section Layer V–obtaining saliency map.

### Comparing the methods for integrating conspicuity maps

**Figure 3:**
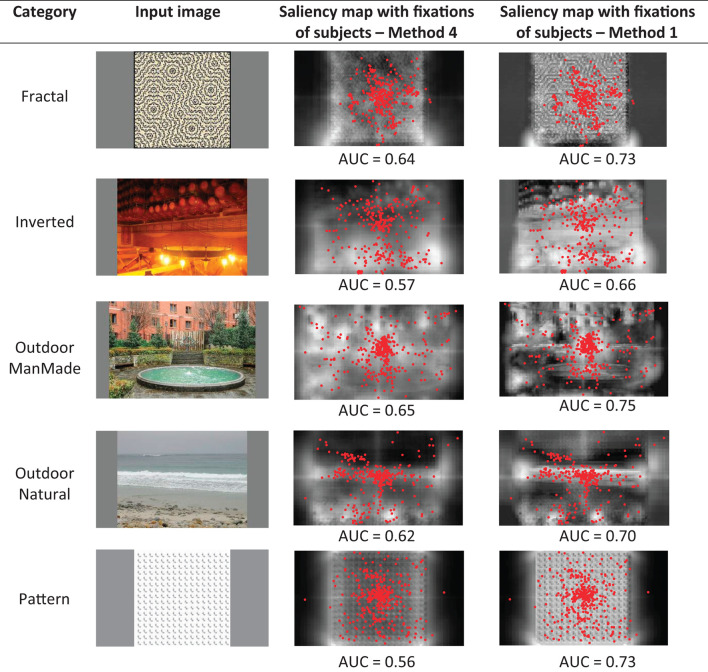
Showing some out of the common images where the first method of obtaining the saliency map is better than the fourth method described in Section Layer V-obtaining saliency map because the conspicuity maps of particular features dominate the other features. The results are shown for the case of applying six center-surround difference maps for each feature.

Before comparing the model results to the other models, we first compare the first and fourth methods of obtaining the saliency map, described in Section Layer V–obtaining saliency map as two different investigated approaches for integrating conspicuity maps. The first method is based on calculating the maximum of conspicuity maps, and the fourth method is based on the weighted summation of the maps. The mean AUC score calculated among all the images of the dataset was higher for the fourth method (AUC = 0.75) compared to the first method (AUC = 0.73). However, for some images, like the example ones shown in Figure 3, the first method performed better, as is seen in the saliency maps and the AUC scores. This shows that, although weighting the maps generally performs better; however, in some images, the conspicuity maps of particular features may dominate the other features, and thus, calculating the maximum of conspicuity maps can make better results for these images.

For example, in Figure 3, in the image of the first row, the edges stand out more than the other features, and therefore, the first method of obtaining the saliency map has resulted in a higher AUC score than the fourth method. In the image of the second row, in specific areas of the image, the intensity or the red-green feature is very bold compared to the other features, and since the first method calculates the maximum of maps over each area, it has gained a higher AUC score. Similarly, in the image of the third row, in the specific areas of the image, the red-green and edge features dominate the other features. In the image of the fourth row, the image contains specific areas of uniform structure, wherein each area, one of the features is bolder compared to other features, making the first method perform better than the fourth method. In the image of the fifth row, the image belongs to the Pattern category, and the pattern used has specific features like edges to be bolder than the other features.

For the rest of the results in this section, the results of the fourth method will be used.

### Comparing different versions of the model to the other models

**Figure 4:**
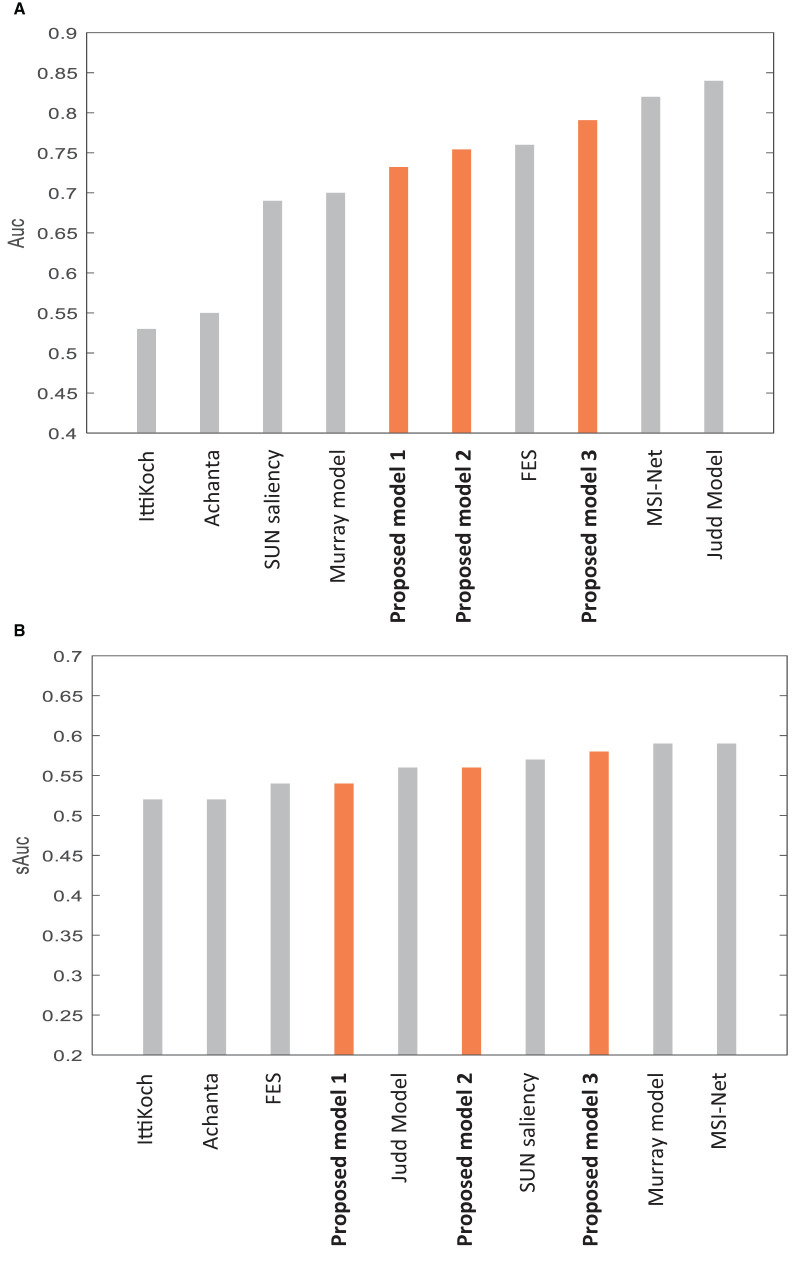
Comparing the performance of our model to several models evaluated on the CAT2000 dataset based on **(A)** mean AUC score and **(B)** mean sAUC score. For more details, refer to the main text.

Many saliency models have been proposed to predict human fixations (Borji and Itti, 2013; Borji et al., 2013; Borji, 2019). We compare our model to several models that used the CAT2000 dataset like ours, and their results are publicly available (http://saliency.mit.edu/results_cat2000.html). We use the metric of AUC-Borji and sAUC (Borji et al., 2013) to compare our model to the models shown in Figure 4, with AUC scores ranging from low to highest reported scores. We use the IttiKoch model (Walther and Koch, 2006) as an extension of the base model of Itti et al. (1998), the Achanta model (Achanta et al., 2009) as one of the most cited models in the frequency domain, and the SUN saliency model (Zhang et al., 2008) and the Fast and Efficient Saliency (FES) model (Tavakoli et al., 2011) as two of the Bayesian-based models. We use the Murray model (Murray et al., 2011) that uses wavelet transform to generate scales like our model. We also use learning-based models, including MSI-Net (Kroner et al., 2020) and the Judd model (Judd et al., 2009). The models of Judd et al. (2009) and Vig et al. (2014), and Zhang and Sclaroff (2016) have the highest AUC score reported on the mentioned website for evaluation on the CAT2000 dataset based on the AUC-Borji metric. Their high AUC is due to setting their parameters according to fixations on trained images. Figure 4 shows our model's mean scores among 20 categories in the dataset, compared to the mentioned models.

In Figure 4, the scores of the three versions of the model described in Section Different versions of the model are compared to other models. In Table 2, the specifications and the scores of the compared models and our model are described. Figure 4 and Table 2 include the models that require no learning as well as the learning-based ones. Our model belongs to non-learning-based models.

**Table 2:** Specifications of the models that were compared to our model in Figure 4, including the non-learning-based models and learning-based ones.

**Model name and references**	**Features**	**Number of scales**	**Method of integrating scales**	**Method of integrating features**	**Learning data**	**AUC**	**sAUC**
IttiKoch (Walther and Koch, 2006)	Low-level: intensity, color, orientations	6	Linear summation	Linear summation, and then estimating the proto-object region based on the salient locations	No	0.53	0.52
SUN saliency (Zhang et al., 2008)	Low-level: intensity, color	4	Data-Driven Bayesian approach	Linear summation	Yes	0.69	0.57
Achanta model (Achanta et al., 2009)	Low-level: color, luminance	The model is frequency-tuned	–	The difference between arithmetic mean pixel value and Gaussian blurred image is calculated for various features	No	0.55	0.52
Judd model (Judd et al., 2009)	Low-level: intensity, color, orientations Mid-level: horizon line High-level: persons, faces	3	Data-Based learning method	Data-Based learning method	Yes	0.84	0.56
Murray model (Murray et al., 2011)	Low-level: intensity, color	Largest dimension of image	–	Euclidean norm of saliency maps of different channels, where each map is calculated based on weighted coefficients of WT of the image. The weights are defined based on CSF	Yes (for setting parameters of the weights defined by contrast	0.70	0.59
FES (Tavakoli et al., 2011)	Low-level: CIELab values	3	Linear summation	Bayesian approach	Yes (for approximating probability values in Bayesian approach)	0.76	0.54
MSI-Net (Kroner et al., 2020)	High-level: image's semantic information	The scales are obtained through three convolutional layers	Encoder-Decoder approach	The feature maps are combined through a convolutional neural network (CNN)	Yes	0.82	0.59
Proposed model 1	Low-level: intensity, color, orientations	6	Weighted summation. The weights are defined based on CSF, calculated locally and globally	Weighted summation. The weights are defined based on the difference between the global maximum and local maxima, calculated locally and globally	No	0.73	0.54
Proposed model 2	Low-level: intensity, color, orientations Medium-level: combination of colors and orientations	6	Same as proposed model 1	Same as proposed model 1	No	0.75	0.56
Proposed model 3 (full version of our model)	Same as proposed model 2	Variable (4, 6, and 10)	Same as proposed model 1 and 2	Same as proposed model 1 and 2	No	0.79	0.58

**Proposed model 1** refers to the version in that we used a fixed number (6) of C–S difference maps, and we did not apply the medium-level features. The AUC results in Figure 4 show that proposed model 1 has high performance (AUC = 0.73) in its related category of non-learning-based models. This high performance indicates that the algorithms used for integrating the information of different scales (described in Section Layer IV–obtaining conspicuity maps) and different features (described in Section Layer V–obtaining saliency map) were efficient in making acceptable results to fit human data.

Proposed model 1 is considered as a baseline to compare to **proposed model 2**, in which we add medium-level features (described in Section Layer I–extracting the features). The AUC score shown in Figure 4 improved by 0.02 (AUC = 0.75) compared to the proposed model 1. The AUC increase suggests that the medium-level features play a role in bottom-up visual attention.

Proposed model 2 is considered as a baseline to compare to **proposed model 3**, in which we add the approach of using variable numbers of 4, 6, and 10 C–S difference maps. We calculate the maximum AUC and sAUC score among the AUC and sAUC scores using 4, 6, and 10 C–S difference maps (described in Section Layer III–creating center-surround difference maps). As is seen in Figure 4, applying variable numbers for C–S difference maps has a considerable effect on improving the AUC results of the model for 0.04 (AUC = 0.79). This suggests that the human visual system may apply different strategies for contrast maps for different images depending on their contents. AUC increase points to better fitting to human gaze data, and gaze is an indicator of human visual attention. In other words, the results indicate a better fit for human attentional behavior.

### The results of using a variable number of contrast maps

**Figure 5:**
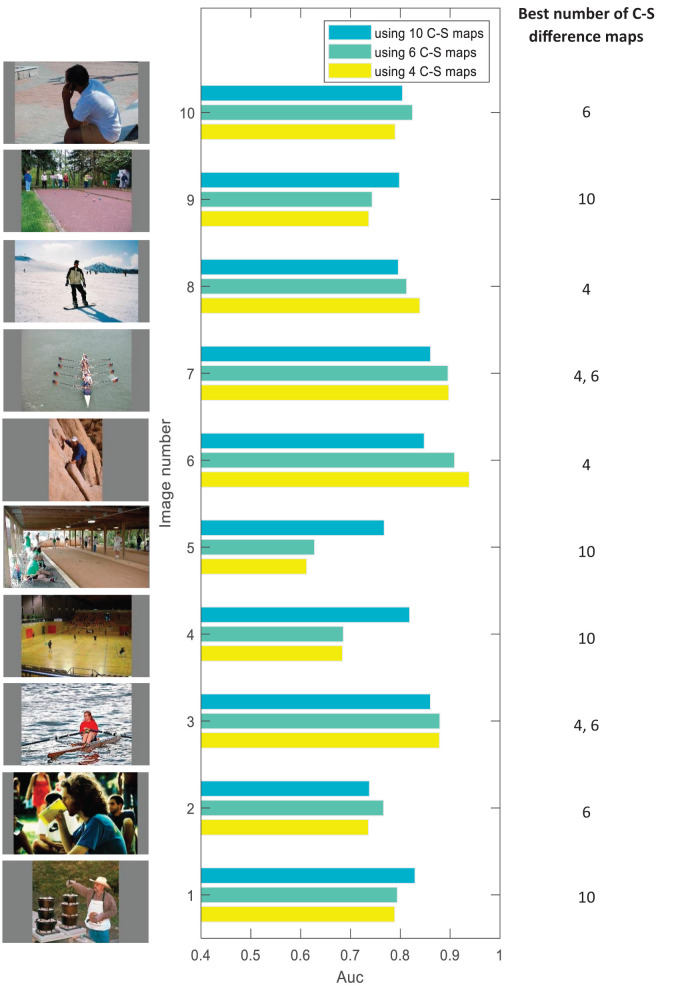
Comparing the AUC results of the model between three different cases of using 4, 6, and 10 C–S difference maps for some sample images from the dataset. The results show that images with different contents require a different number of C–S difference maps to result in high performance for the model.

**Figure 6:**
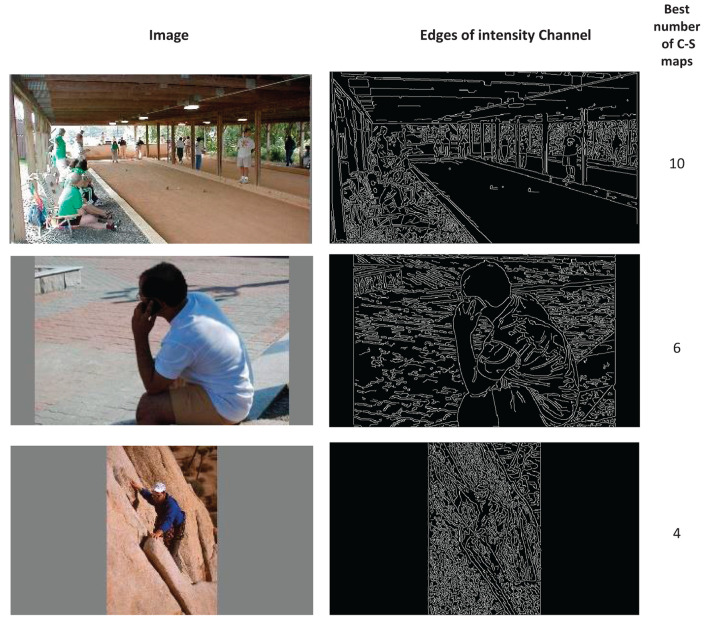
The results for edge detection of sample images of Figure 5. The images are shown as examples for the best number of 4, 6, and 10 for C–S difference maps. The edge detection shows a rough estimation of the detailed and coarse content of the images. The edges are obtained for the intensity channel of the images.

To make our proposal about using a variable number of C–S difference maps more visible, in Figure 5, we compare the model results between three cases of using 4, 6, and 10 C–S difference maps for some sample images from the dataset. Referring to the description in Section Layer III–creating center-surround difference maps, if an image has mainly detailed content, using 4 C–S maps would probably lead to better performance than 6 or 10 C–S maps. If an image has detailed and middle-coarse content, using 6 C–S maps would be better, and if an image has detailed, middle-coarse and coarse content, using 10 C–S maps would be better. We may say that the more big structures an image has, the more coarse content it would have, and a higher number of C–S maps would probably lead to better performance. The edge density over the images may give us a rough estimation of the detailed and coarse content of the images. Figure 6 shows the results for edge detection of sample images in Figure 5 as examples for the best number of 4, 6, and 10 for C–S difference maps. However, finding a direct relation between the best number of C–S maps and the image content is not straightforward because the image content cannot be described by a single factor; but rather by several factors such as edge densities, frequency content, histogram of intensities, the number and the size of objects in the image, and image texture.

Based on the results in Figures 4, 5, we propose that finding a mechanism to apply in the models to get information about the image content and producing the C–S difference maps based on the image content can make the models more efficient. In connection with the factors mentioned above to describe the image content, some approaches like frequency analysis, calculating image crowdedness, object detection, and edge detection can be investigated. Another approach that may be useful is a hierarchical segmentation of the image to determine the number of big and small structures in the image.

**Figure 7:**
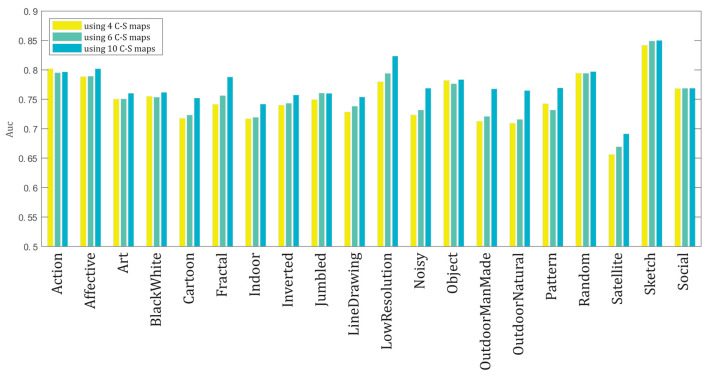
The mean AUC scores of the model for the 20 categories of the CAT2000 dataset for the three cases of applying 4, 6, and 10 C–S difference maps in the model.

In Figure 7, the AUC results of the model for the 20 categories of the dataset are shown for the three cases of applying 4, 6, and 10 C–S difference maps. The AUC score is calculated in each category by averaging the AUC scores for the 100 images of the associated category for each case of using 4, 6, and 10 maps. The model has the highest performance for the Sketch category, probably because of the simpler structure of the images in this category, and it has the lowest performance for the Satellite category, probably because of the very low resolution and bad quality of the images in this category. We use approximation information of the wavelet transform of the image, and not the detailed information of WT, while due to the very low quality of the images in the satellite category, more detailed information of the image is required to make a high performance by the model.

In each category, dependent on the overall content of the images in that category, the mean AUC score for that category may be higher for the case of applying 4, 6, or 10 C–S difference maps. As discussed in the results of Figure 5, relating the best number of C–S maps and the image content requires deeper investigation. However, the results of some categories may be interpreted based on their image types. For example, in the LowResolution category, because of the images' low resolution, the images' detailed content is blurred; thus, the salient areas may be obtained mostly based on the coarse content of the images. Therefore, using 10 C–S difference maps would lead to better performance for most of the images in this category, and a clearer difference is visible between the AUC results of using 10 C–S maps compared to 4 and 6 C–S maps. Similarly, in the category Noisy, because of the presence of noise in the images, and in the category Satellite, because of the images' low quality, the detailed content of the images cannot be detected easily. Therefore, similar to the category LowResolution, using 10 C–S maps would lead to better performance in these two categories.

## Discussion

Recently, the focus of saliency models on investigating bottom-up attention has decreased, while there are still open questions about applying bottom-up mechanisms. These questions include what features should be applied, how to obtain the contrasting areas, and how to integrate the information of different scales and features of the image. Here we present a bottom-up saliency model to address these questions and improve previous models. We present our improvements in different versions of the model as proposed models 1, 2, and 3 and compare their performances to show the effect of each improvement separately.

First, in proposed model 1, we integrate the information of different scales (described in Section Layer IV–obtaining conspicuity maps) and features (described in Section Layer V–obtaining saliency map) based on their weighted sum. The weight of contrast maps of different scales for a specific feature depends on the importance of the spatial frequency information of that map, which we calculate it using the contrast sensitivity function. This function of the visual system is not considered in so many models. The authors (Murray et al., 2011; Buzatu, 2012; Wang et al., 2018) use this function in their model; however, they do not apply it for weighting different scales of the feature maps. In addition, we utilize both global and local weighting on the contrast maps of the scales.

The weights of the conspicuity maps of different features are defined based on a preliminary study testing 12 different variations of the integration methods and applying four different methods among them. Finally, we choose the method with the best performance among these four methods. The weights are defined based on the difference between the global maximum and local maxima in the related conspicuity map. This weighting method for features is applied in a similar way in some other models (Itti and Koch, 2001; Frintrop et al., 2007). In contrast to them, we calculate the weights of conspicuity maps to present both the local and global contrast of an area. Comparing our model 1 to other models (Figure 4, Table 2) based on AUC and sAUC metrics suggests that the integration mechanisms applied in the model perform better than other non-learning-based models to make the model fit human data.

Furthermore, we compare our weighting method for integrating the conspicuity maps of different features with the method of calculating the maximum of conspicuity maps. The comparison results show that overall, the mean AUC score for the dataset images is higher for the weighting method than for calculating the maximum of conspicuity maps. However, as shown in the results section for some sample images (Figure 3), in some images, the maximum method performs better than the weighting method. This shows that, although the weighting method leads to better overall performance, in some images, particular features may be too salient compared to other features in various areas of the image, and this overcoming saliency causes calculating the maximum of conspicuity maps leads to better results.

Second, in proposed model 2, we extend proposed model 1 so that, in addition to the low-level features commonly used in other models, we apply medium-level features based on the combination of color features with orientations (described in Section Layer I–extracting the features). The increased performance of proposed model 2 compared to proposed model 1 (Figure 4) suggests that the medium-level features also play a role in bottom-up visual attention behavior.

Third, in proposed model 3, we extend proposed model 2 so that we apply a variable number of C–S difference maps instead of a fixed number as common in other models (described in Section Layer III–creating center-surround difference maps). We discuss that human visual attention does not act the same for different images with different contents. To investigate this idea, we implemented the model using 4, 6, and 10 contrast maps for each feature. The chosen numbers refer, respectively, to the low, medium, and a high number of contrast maps. We calculated the AUC score by computing the maximum score among the scores of using 4, 6, and 10 C–S difference maps. The considerably increased performance (AUC by 0.04 and sAUC by 0.02) of proposed model 3 compared to proposed model 2 in Figure 4 suggests that our proposal about applying a variable number of contrast maps can make the model more fitting to human data. This suggests that human visual attention may not act the same for different types of images, and the proper number of contrast maps for each image depends on the amount of detailed and coarse content in the image, as is shown for some sample images of the dataset in Figure 5. In addition, comparing the results of applying different numbers of contrast maps for all 20 categories of the CAT2000 dataset (Figure 7) confirmed our proposal. The center-surround difference mechanism is the base and essential mechanism of visual attention, and this improvement was the most important improvement made in our model.

We propose to apply a mechanism in the model to get information about the image content and adapt the number of C–S difference maps to the image content, as discussed in Section The results of using a variable number of contrast maps. This adaptation mechanism can be used in the saliency models to improve their performances. Furthermore, the adaptation mechanism would minimize the additional computations due to applying a variable number of C–S maps. The detailed and coarse content of the images may be roughly estimated by the images' edge map, as shown in Figure 6 for some sample images. However, further investigation is required to find the factors that can be used to describe image content and be relative to the best number of C–S maps. The factors such as edge densities, the ratio of low and high frequencies in the image, histogram of intensities, image crowdedness, and image texture could be investigated. Moreover, hierarchical segmentation can be applied to the image to extract the amount of big and small structures to indicate the amount of detailed and coarse content in the image. The analysis of image content could be improved even further to make it more adaptive in the way that a different number of C–S maps is applied to distinct image regions with different structures. Finding an analyzing method to adapt the number of C–S maps based on the image content is the scope of future study.

We validated our model's performance to describe human data by applying it to the CAT2000 dataset and measuring its performance based on the AUC and sAUC metrics. Comparing the results of our model to the other models that used the same dataset (Figure 4) shows that our model has a high performance in the category of non-learning-based models, with the AUC of 0.73, 0.75, and 0.79, and sAUC of 0.54, 0.56, 0.58, respectively, for model versions 1, 2, and full version 3. The high performances of saliency models have been reported mostly for learning-based methods. The high performance of these learning-based models is related to the fact that they predict the fixations on the images by setting their parameters according to fixations on trained images. These learning-based models look promising; however, they usually require large human datasets to perform well. Also, learning-based methods may not be perfect for showing attention mechanisms. Usually, they show us what would be attended by the visual system but not how or why it may be attended. The advantage of our model is that it shows high performance according to AUC and sAUC metrics based on the applied mechanisms, and it does not have the complexity of learning-based methods that need to learn human data. We suggest that our proposed mechanisms can be added to the learning-based models to make them achieve higher performance and fit better with human data.

## Conclusion

We proposed a saliency model that addresses some mechanisms of visual attention behavior that other models do not take into account. We defined three versions of the model to investigate the effect of each improvement separately. As a first improvement, in proposed model 1, we applied a weighted summation method for integrating the information of different scales and different features, defining the weights according to the contribution of each component, and presenting both the local and global saliency of the image. The model's high performance compared to the other models indicates that the integration methods were efficient. Furthermore, we compared the weighted summation method for combining the conspicuity maps of different features with the method of calculating the maximum of conspicuity maps. The comparison showed that although the weighted summation method leads by average to better performance, however, in some images where particular features dominate the other features, calculating the maximum of conspicuity maps can make better results. Second, in proposed model 2, in addition to the common low-level features, we added the medium-level features to proposed model 1. The increased AUC and sAUC of proposed model 2 compared to model 1 suggests that medium-level features may play a role in the behavior of visual attention. Third, and most importantly, in proposed model 3, instead of the common approach of using a fixed number of C–S difference maps, we added the mechanism of variable numbers of these maps to proposed model 2, proposing that human visual attention performs differently for different images. The increased AUC and sAUC of proposed model 3 compared to model 2 confirms our proposal about the center-surround mechanism. The mechanism of a variable number of C–S difference maps can improve further to make it adaptive to the image content.

## Data availability statement

The original contributions presented in the study are included in the article/supplementary material, further inquiries can be directed to the corresponding authors.

## Author contributions

SN contributed to the conceptualization of the study, methodology, writing the first draft of the manuscript, and visualization. AF contributed to the supervision of the study. SR and MD contributed to manuscript revision. All authors approved the submitted version.
